# A clinical trial on 3D CT scan and polysomnographyc changes after rapid maxillary expansion in children with snoring

**DOI:** 10.1016/j.bjorl.2022.04.004

**Published:** 2022-05-20

**Authors:** Rita Catia Brás Bariani, Renato Bigliazzi, Fauze Ramez Badreddine, Lucia Hatsue Yamamoto, Sergio Tufik, Gustavo Moreira, Reginaldo Raimundo Fujita

**Affiliations:** a Universidade Federal de São Paulo (Unifesp), Departamento de Otorrinolaringologia e Cirurgia de Cabeça e Pescoço, São Paulo, SP, Brazil; b Private Pratice in Orthodontics, São Paulo, SP, Brazil; c Universidade Federal de São Paulo (Unifesp), Departamento de Psicobiologia, São Paulo, SP, Brazil

**Keywords:** Sleep apnea, obstructive, Palatal expansion technique, Imaging, three-dimensional, Craniofacial abnormalities, Sleep apnea syndromes

## Abstract

**Objective:**

The present prospective clinical study aimed to investigate the effects of rapid maxillary expansion on the airway, correlating airway volumes obtained on multi-slice computed tomography and polysomnography assessment of oxygen saturation and apnea/hypopnea index.

**Methods:**

Twenty-four patients (11 with obstructive sleep apnea and 13 with residual snoring, mean age 10.0 (1.8), were enrolled in the study. Each patient underwent multislice computed tomography and nocturnal polysomnography before rapid maxillary expansion and after removal of maxillary expansion after six months. Airway regions were segmented, and volumes were computed.

**Results:**

The increase in oropharyngeal volume was significant in both groups. Oxygen saturation and apnea/hypopnea index were not statistically significant. No correlation was found between total airway volume, oxygen saturation, and apnea/hypopnea index changes between the time points examined.

**Conclusions:**

This study showed that when rapid maxillary expansion is performed in individuals with sleep-disordered breathing, there were statistically significant differences in oropharyngeal volume between pre- and post-rapid maxillary expansion, but there was no correlation between oxygen saturation values and oropharyngeal volume increase.

**Level of evidence:**

The article is classified as Evidence Level 3 (Three).

## Highlights

• RME should be a treatment option in children with SBT and maxillary hypoplasia.
• The oropharynx volume increased after rapid maxillary expansion.
• Differences in oropharyngeal volume between pre- and post RME in individuals with SDB.

## Introduction

Obstructive Sleep Apnea Syndrome (OSAS) is a relatively common disorder in childhood that causes behavioral problems,^1^ attention and learning deficits, evidence of neuronal brain damage, increased cardiovascular risk profile, and poor quality of life.^2^, ^3^, ^4^, ^5^ Habitual snoring rates vary significantly in the literature, depending on the definition adopted, the age group studied, and the questionnaire employed. The prevalence of chronic snoring found among Brazilian children was 27.6%, while 0.8% reported apnea, 15.5% described daytime mouth breathing, and 7.8% complained of excessive daytime sleepiness.^6^

The most common otorhinolaryngological disorder in children with OSAS is adenoid and tonsil hypertrophy, accompanied by allergic rhinitis, snore, mouth breathing, episodes of apnea, and restless sleep difficulties in swallowing saliva at night, as well as in chewing/swallowing, and postural changes.^7^

Adenotonsillectomy (AT) is the recommended first-line treatment for OSAS in children with adenotonsillar hypertrophy.^8^ However, a significant number of children with OSAS undergoing AT exhibit residual SDB after surgery.^9^ Although OSAS improved postoperatively, the proportion of patients who had residual OSAS ranged from 13% to 29% in low-risk populations to 73% when obese children were included, and stricter polysomnographic criteria were used.^10^ Possible factors influencing AT failure are patients with comorbidities, craniofacial anomalies, and nasal obstruction.^11^, ^12^

Children with craniofacial anomalies with OSAS have a high-arched palate, increased mandibular plane angle, constricted upper and lower arches, and retrognathic mandible. Maxillary constriction deficiency plays a role in the etiology of severe respiratory disorders such as OSAS in growing individuals.^12^, ^13^ Rapid Maxillary Expansion (RME) improves the nasal airway patency and effectively treats obstructive sleep apnea in children with maxillary constriction.^14^

Upper airway three-dimensional analysis and evaluation have received significant attention in the literature due to the more straightforward assessment and advantages provided by newer three-dimensional imaging techniques. Upper airway evaluation includes nasopharyngoscopy, cephalometry, multi-slice Computed Tomography (CT), Magnetic Resonance Imaging (MRI), and Cone-Beam Computed Tomography (CBCT) for visualization and measurement of the pharyngeal airway volume.^15^ There are differences in upper airway morphology and function between supine and upright positions during the acquisition of images generated by CT and CBCT for upper airway assessment.^16^ The airway of OSA patients was smaller when the patients were in a supine compared with an upright position. Airway changes in obstructive sleep apnea patients associated with a supine versus an upright position examined using cone-beam computed tomography.^17^

This prospective study aimed to investigate the effects of RME in children with residual snoring and obstructive sleep apnea after adenotonsillectomy, by correlating airway volumes using Multi-Slice Computed Tomography (MCT) and polysomnography.

## Methods

### Study design

This study is a prospective clinical trial of an orthodontic intervention registered in the Brazilian Clinical Trials Registry (ReBEC): RBR-463by. Patients with residual snoring and transverse maxillary deficiency submitted to adenotonsillectomy two or more years earlier underwent the following exams before and after RME treatment: orthodontic documentation, nasofibroscopy, polysomnography, and neurocognitive tests. The participants were recruited from patients attending the Pediatric Otorhinolaryngology Outpatient Clinic of Escola Paulista de Medicina at Universidade Federal de São Paulo (UNIFESP). The Institutional Review Board approved the study under number 0698011806/2017. All parents gave written informed consent after receiving information about the protocol, monitoring, and treatment proposed. This clinical trial was carried out from December 2017 to March 2021.

### Recruitment

The caretakers of three hundred patients aged 5–12 years old, who had undergone adenotonsillectomy in the previous four years at Hospital São Paulo, were contacted by telephone, and those who continued to present snoring symptoms at least five times a week were recruited. They were asked to attend a consultation with an otorhinolaryngologist who performed a laryngeal nasofibroscopy examination. Patients who required further surgery due to adenoid and lingual tonsillar hypertrophy were excluded. Patients with heart and neuromuscular diseases, craniofacial malformations, chromosomal syndromes, dental changes that precluded RME (loss of teeth, dental caries, and periodontal disease), and users of neurological drugs were excluded. Therefore, the experimental group consisted of 24 patients with a mean age of 10.0 (1.8) with maxillary constriction, treated with Hyrax palatal expanders.

### RME clinical protocol

Following upper and lower alginate impressions, a Hyrax expander was constructed with two bands, palatal stainless-steel bars with a 1.0-mm diameter, and an expanding screw (Morelli — Dental Morelli, São Paulo, Brazil) with stainless steel extensions soldered to the palatal surfaces of each band. Each quarter turn activation of the screw was equivalent to 0.25 mm. One week after the expander was cemented in situ; the screw was activated (six turns) by an orthodontist to start the expansion. The parents were instructed to activate the screw two turns per day (0.5 mm), and the patients were recalled weekly during the expansion period (2–3 weeks). The expansion was stopped when the palatal cusp of the upper molars was touching the buccal cusp of the lower molars. The device then remained in place for six months to allow the formation of bone in the midpalatal suture. An experienced orthodontic consultant blinded to this research performed all clinical treatments.

### Polysomnography

All patients underwent standard in-laboratory full-night polysomnography using a digital polysomnography machine (Embla N7000®, Embla Systems, Inc., Broomfield, CO, USA). Nasal airflow was recorded using both a nasal cannula/pressure transducer system and a thermocouple. The criteria adopted for sleep-wake scoring met the accepted international standards while scoring respiratory events followed the American Academy of Sleep Medicine^18^ criteria. The following sleep parameters were assessed: sleep latency; rapid eye movement (REM) sleep latency; sleep efficiency; all sleep stages (N1, N2, N3, and REM) calculated as a percentage of total sleep time; respiratory events (obstructive apnea, hypopnea, respiratory effort-related arousal, and respiratory disturbance index score); the number of arousals, and mean oxygen saturation. Residual OSA was defined as an Obstructive Apnea-Hypopnea index >1/h in addition to percutaneous oxygen saturation (SpO2) <92%.^19^

### CT image acquisition and analysis

All subjects underwent two low-dose CT scans in supine position. The CT images were obtained before (T1) and after RME (T2) with approximately six months between times. All CT scans were performed in the Department of Diagnostic Imaging of the institution, using a multi-slice device (Philips® Brilliance CT scanner 64 channels).

Raw data obtained from CT scanning were exported as Digital Imaging and Communications in Medicine format (DICOM) and reconstructed into a specific software (Dolphin Imaging 11.7 Premium software: Dolphin Imaging and Management Solutions, Chatsworth, Calif).

**Figure 1:**
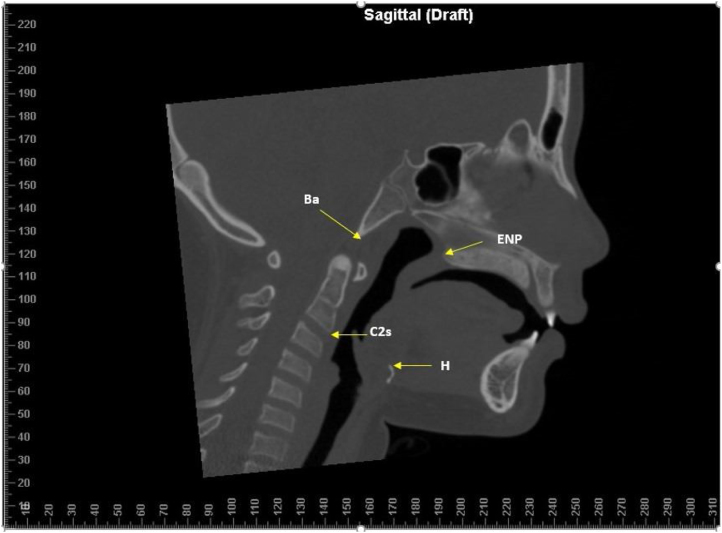
Anatomical points. Basion (Ba): Point located in the lower portion on the anterior margin of the foramen magnum; C2 (Second cervical vertebra): Lower anterior point of the C2 vertebra; Posterior Nasal Spine (PNS): Tip of the posterior nasal spine of the palatine bone at the junction of the soft and hard palate; Hyoid (H): Uppermost point of the Hyoid bone.

**Figure 2:**
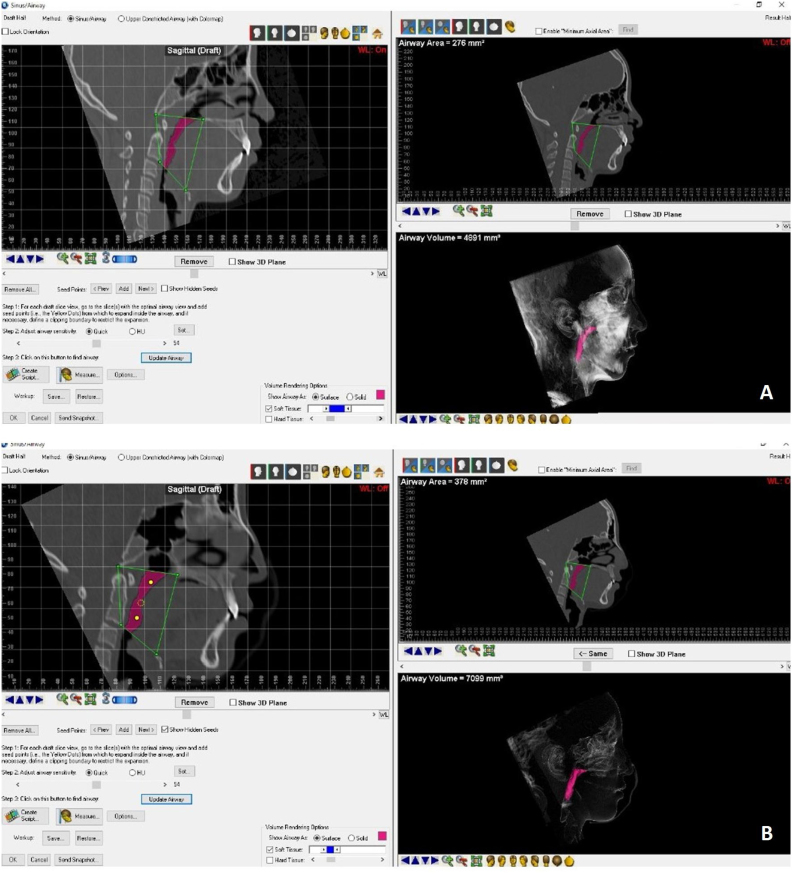
Measurement of the oropharynx airway volume pre-RME (A) and post-RME (B), using the Dolphin imaging software.

Then, on multiple planar reconstruction images, they were reoriented using the Frankfort horizontal plane (FH plane) as the horizontal reference plane.^20^ In profile view, FH plane connects the highest point of the opening of the external auditory canal with the lowest point on the lower margin of the orbit, used to orient a human skull or head so that the plane is horizontal and represents the natural head position. The images were evaluated in three views (sagittal, coronal, and axial). Measurements were taken to verify changes in volume after RME; for that purpose, specific anatomical points were defined as described in Table 1 and Fig. 1. Once the anatomical points of interest were defined, the methodology used to verify the pre- and post-RME volume followed the parameters described in Fig. 2.

**Table 1:** Anthropometric parameters in children at baseline and 6-months after treatment (*n* = 24).

Variables		Baseline	6-months after treatment	Student *t-*test
Age (years)	Min – Max	6.1 – 12.7	6.8 – 14.6	
	Mean (SD)	10.0 (1.8)	11.3 (1.9)	*p* < 0.001
Weight (Kg)	Min – Max	19 – 61	21 – 70	
	Mean (SD)	36.0 (9.0)	40.9 (10.5)	*p* < 0.001
Height (meters)	Min – Max	0.97 – 1.55	1.10 – 1.60	
	Mean (SD)	1.36 (0.14)	1.46 (0.12)	*p* < 0.001
BMI (*Z-*score for age)	Min – Max	−2.13 – 5.21	−3.09 – 3.28	
	Mean (SD)	0.87 (2.16)	0.37 (1.76)	*p* = 0.098
Sex	Female	*n* = 8 (33.3%)		
	Male	*n* = 16 (66.7%)		

BMI, Body Mass Index; Min, Minimum; Max, Maximun; SD, Standard Deviation.

In sagittal view, the points Ba (Basion), Posterior Nasal Spine (PNS), Hyoid (H), and C2i (inferior anterior edge of the C2 vertebra) were selected. After delimitation by these selected 4 points, the volume of the oropharynx before and after RME was measured, using specific software tools.

### Statistical analysis

All data were analyzed using SPSS Statistics for Windows. Data are presented as means and standard deviations for continuous variables and frequencies for categorical variables. The repeated measures ANOVA test with time interaction (Pre-RME vs. Post-RME) and group (Primary Snoring vs. OSA) was used to respond to the research objectives. The independent *t*-test was used to compare the two groups (Primary Snoring and OSA) before and after RME. The paired-sample *t*-test was applied to evaluate the significance of Pre- and Post-RME differences in the total sample. Cohen’s d was used, considering the following criteria to characterize the differences *d* = 0.2 (small effect), *d* = 0.5 (moderate effect), and *d* ≥ 0.8 (large effect) between Pre- and Post-RME. Pearson’s Correlation Coefficient test was used to identify the correlations between the total score of OSA-18 and OAHI before and after RME. The differences were statistically significant when the significance value was lower than 0.05 (*p* < 0.05). Measurements were repeated by the same observer (intra-observer error analysis) and by a second observer (inter-observer error analysis) to assess the study error of the oropharyngeal volume.

## Results

### Definition of groups

The 24 patients in the sample were divided into two groups based on Pre-RME AHI values, considering the following criteria: Primary Snoring Group — OAHI < 1 (*n* = 13); OSA Group: OAHI ≥ 1 (*n* = 11).

Thus, the analyses were performed on the total sample (*n* = 24) and separately for the Primary Snoring group (*n* = 13) and the OSA group (*n* = 11).

The sample comprised 24 patients, primarily males (*n* = 16, 66.7%), aged between 6.1 and 12.7 years before RME. The mean age was 10.0 years, and the standard deviation was 1.8 years. On average, they weighed 36.0 kg and were 1.36 m high. As expected, age, weight, and height significantly increased between the Pre-RME and Post-RME periods (*p* <  0.001). As for age-adjusted BMI (Z-score for age), the mean decreased from 0.87 (SD = 2.16) before RME to 0.37 (SD = 1.76) after RME, however the differences were not statistically significant (*p* =  0.098) (Table 1).

### Polysomnography

The results of the characterization of polysomnography values before and after RME are presented in Table 2. The time*group interaction values were non-significant (*p* > 0.05) for all polysomnography variables, indicating no significant differences between groups regarding Pre-RME and Post-RME evolution.

**Table 2:** Characterization and comparison of polysomnography data before and after RME.

Variables	Group	Pre-RME M (SD)	Post-RME M (SD)	Difference in dimension (d)	p Pre-Post	p interaction group*time
AHI	**Total**	1.49 (1.32)	2.07 (2.85)	0.22	0.289	
	Primary snoring	0.83 (1.08)	1.87 (3.65)	0.32	0.266	0.352
	OSA	2.26 (1.19)	2.33 (1.41)	0.05	1.000	
	**group p**	**0.005**	0.710			
OAHI	**Total**	1.07 (1.09)	1.23 (1.47)	0.11	0.570	
	Primary snoring	0.37 (0.30)	1.08 (1.67)	0.46	0.124	0.095
	OSA	1.89 (1.12)	1.42 (1.22)	0.43	0.163	
	**group p**	**<0.001**	0.599			
CAHI	**Total**	0.43 (0.70)	0.73 (1.54)	0.21	0.338	
	Primary snoring	0.48 (0.84)	0.76 (2.03)	0.16	0.585	0.983
	OSA	0.36 (0.52)	0.68 (0.56)	0.47	0.205	
	**group p**	0.684	0.904			
RDI	**Total**	1.60 (1.39)	1.98 (2.82)	0.14	0.489	
	Primary snoring	0.94 (1.13)	1.91 (3.64)	0.30	0.300	0.236
	OSA	2.37 (1.29)	2.07 (1.30)	0.21	0.431	
	**group p**	**0.008**	0.895			
Basal SpO_2_	**Total**	96.43 (0.94)	96.29 (1.37)	0.12	0.508	
	Primary snoring	96.71 (0.87)	96.82 (0.89)	0.14	0.629	0.213
	OSA	96.11 (0.96)	95.60 (1.62)	0.33	0.309	
	**group p**	0.124	0.032			
Mean SpO_2_	**Total**	95.60 (1.26)	95.12 (1.67)	0.37	0.104	
	Primary snoring	96.00 (1.18)	95.57 (1.66)	0.38	0.191	0.930
	OSA	95.12 (1.22)	94.54 (1.57)	0.38	0.346	
	**group p**	0.087	0.146			
Min SpO_2_	**Total**	90.83 (3.03)	91.00 (2.75)	0.06	0.749	
	Primary snoring	92.54 (1.81)	92.15 (1.82)	0.16	0.568	0.243
	OSA	88.82 (2.99)	89.50 (3.10)	0.25	0.331	
	**group p**	**0.001**	**0.018**			
DI	**Total**	1.28 (1.25)	1.54 (1.60)	0.19	0.315	
	Primary snoring	0.63 (0.92)	0.79 (0.98)	0.15	0.594	0.620
	OSA	2.05 (1.17)	2.52 (1.75)	0.28	0.419	
	**group p**	**0.003**	**0.007**			

Results presented in the form: mean (standard deviation).

RME, Rapid Maxillary Expansion; Pre, Baseline; Post, 6-months after treatment; M, Mean; OSA, Obstructive Sleep Apnea; AHI, Apnea-Hypopnea Index; SpO_2_%, Average Overnight Arterial Oxygen Saturation (basal, mean, min: minimum); OAHI, Obstructive Apnea/Hypopnea Index; CAHI, Central Apnea/Hypopnea Index; RDI, Respiratory Disorder Index, saturation index; DI, Desaturation Index.

### Oropharynx volume

#### Measurement error

The significance values of the Wilcoxon tests for paired samples for comparison of the initial measurements and replicates were non-significant (*p* >  0.05) in both observers, indicating no statistically significant differences between the initial measurements and replicates. Intraclass Correlation Coefficient (ICC) values were above 0.99, indicating excellent measurement consistency levels. Together, the Wilcoxon Test and ICC results assure the non-existence of systematic or random error, demonstrating the consistency and reliability of measurements.

#### Oropharynx volume

The results in Table 3 show that, in the total sample, there was a statistically significant increase (*d* = 0.72, *p* =  0.002) in the oropharynx volume after RME (*M* = 10631.71), compared to that observed before RME (*M* = 8656.09). Concerning the evolution in each group, the increase was significant in the Primary Snoring group (mean increased from 11098.34 to 13723.33, *d* = 0.82, *p* =  0.012) and close to statistical significance in the OSA group (mean increased from 5769.80 to 6977.98, *d* = 0.61, *p* =  0.072). The p-value of the time*group interaction was not significant (*p* =  0.218), indicating no significant differences between groups regarding the Pre-RME — Post-RME evolution. Regarding the comparison between groups, the mean oropharyngeal volume was higher in the Primary Snoring group than in the OSA group; however, the differences were not significant before RME (*p* =  0.308) or after RME (*p* = 0.275).

**Table 3:** Characterization and comparison of oropharyngeal volume before and after RME.

Variables	Group	Pre-RME M (SD)	Post-RME M (SD)	Difference dimension (d)	p Pre-Post	p interaction group*time
Volume	**Total**	8656.09 (12498.82)	10631.71 (14793.89)	0.72	**0.002**	
	Primary Snoring	11098.34 (16673.82)	13723.33 (19683.44)	0.82	**0.012**	0.218
	OSA	5769.80 (2961.41)	6977.98 (3367.60)	0.61	0.072	
	**p groups**	0.308	0.275			

Results presented as mean (standard deviation).

RME, Rapid Maxillary Expansion; M, Mean; SD, Standard Deviation; OSA, Obstructive Sleep Apnea.

#### Saturation

The results in Table 4 show no significant differences between Pre-RME and Post-RME in any of the saturation parameters, neither in the total sample nor in any of the two groups (*p* >  0.05). The non-significant time*group interaction (*p* >  0.05) indicates no significant differences between groups regarding the Pre-RME — Post-RME evolution. Significant differences were also identified after RME in mean wakeful Sat. (*p* =  0.040), REM mean Sat. (*p* =  0.040), and NREM mean Sat. (*p* =  0.034): the mean values of these variables were higher in the Primary snoring group than in the OSA group.

**Table 4:** Characterization and comparison of saturation values before and after RME.

Variables	Group	Pre-RME M (SD)	Post-RME M (SD)	Difference dimension (d)	p Pre-Post	p interaction group*time
Mean Sat. awake	**Total**	96.49 (0.94)	96.20 (1.29)	0.26	0.213	
	Primary snoring	96.81 (0.82)	96.69 (0.82)	0.15	0.593	0.412
	OSA	96.12 (0.96)	95.62 (1.53)	0.35	0.277	
	**p groups**	0.071	**0.040**			
Mean Sat. REM	**Total**	95.90 (1.31)	95.47 (1.37)	0.34	0.108	
	Primary snoring	96.32 (1.18)	95.99 (0.86)	0.48	0.107	0.697
	OSA	95.39 (1.32)	94.86 (1.64)	0.31	0.328	
	**p groups**	0.081	**0.040**			
Mean Sat. NREM	**Total**	95.55 (1.28)	95.17 (1.31)	0.33	0.120	
	Primary snoring	95.95 (1.23)	95.68 (0.95)	0.28	0.333	0.597
	OSA	95.08 (1.22)	94.56 (1.46)	0.37	0.246	
	**p groups**	0.100	**0.034**			
Total desat	**Total**	8.00 (7.66)	12.38 (17.08)	0.27	0.195	
	Primary snoring	3.69 (4.92)	9.54 (20.00)	0.31	0.288	0.636
	OSA	13.09 (7.29)	15.73 (12.98)	0.21	0.498	
	**p groups**	**0.001**	0.388			
SDI	**Total**	1.29 (1.24)	2.00 (2.78)	0.27	0.197	
	Primary snoring	0.65 (0.91)	1.64 (3.48)	0.31	0.292	0.566
	OSA	2.05 (1.17)	2.42 (1.69)	0.22	0.474	
	**p groups**	**0.003**	0.506			
SDI REM	**Total**	2.07 (3.38)	2.99 (4.31)	0.21	0.313	
	Primary snoring	0.86 (1.34)	1.85 (3.13)	0.27	0.354	0.936
	OSA	3.50 (4.46)	4.34 (5.22)	0.16	0.607	
	**p groups**	**0.054**	0.163			
SDI NREM	**Total**	1.06 (1.06)	1.65 (2.73)	0.24	0.251	
	Primary snoring	0.58 (0.94)	1.52 (3.62)	0.29	0.315	0.474
	OSA	1.62 (0.94)	1.81 (1.17)	0.17	0.590	
	**p groups**	**0.014**	0.805			
OAHI SUPINE	**Total**	2.77 (4.51)	1.96 (3.05)	0.16	0.451	
	Primary snoring	2.52 (5.66)	1.71 (3.72)	0.12	0.676	0.994
	OSA	3.05 (2.85)	2.25 (2.15)	0.36	0.266	
	**p groups**	0.781	0.672			
OAHI NON-SUPINE	**Total**	1.27 (1.25)	1.67 (3.02)	0.13	0.592	
	Primary snoring	0.66 (1.05)	2.03 (4.05)	0.36	0.253	0.103
	OSA	1.99 (1.11)	1.26 (1.30)	0.57	0.086	
	**p groups**	**0.007**	0.554			

Results presented as mean (standard deviation).

RME, Rapid Maxillary Expansion; OSA, Obstructive Sleep Apnea; REM, Rapid Eye Movement; NREM, Non**-Rapid** Eye Movement sleep; SDI, Sleep Desaturation Index; OAHI SUPINE, Apnea Index + Obstructive Hypopnea Supine Position; OAHI NON-SUPINE, Apnea Index + Obstructive Hypopnea Non-Supine Position.

#### Correlation of saturation with the oropharyngeal volume

The correlations between saturation values and oropharyngeal volume were close to zero and non-significant (*p* > 0.05), both before RME and after RME (Table 5).

**Table 5:** Correlation of saturation values with oropharyngeal volume, before and after RME.

Saturation	Oropharyngeal volume	
	Before RME	After RME
Mean Sat. awake	*R* = −0.207^a^	*R* = 0.062^a^
Mean Sat. REM	*R* = −0.245^a^	*R* = −0.141^a^
Mean Sat. NREM	*R* = −0.253^a^	*R* = −0.040^a^
Total desat	*R* = −0.180^a^	*R* = −0.198^a^
SDI	*R* = −0.148^a^	*R* = −0.195^a^
SDI REM	*R* = −0.049^a^	*R* = −0.164^a^
SDI NREM	*R* = −0.202^a^	*R* = −0.178^a^
OAHI SUPINE	*R* = −0.136^a^	*R* = −0.147^a^
OAHI NON-SUPINE	*R* = −0.108^a^	*R* = −0.139^a^

*R*, Pearson Correlation Coefficient; * *p* < 0.05 (coefficients significantly different from zero).

RME, Rapid Maxillary Expansion; REM, Rapid Eye Movement; SDI, Sleep Desaturation Index; OAHI SUPINE, Apnea Index + Obstructive Hypopnea Supine Position; OAHI NON-SUPINE, Apnea Index + Obstructive Hypopnea Non-Supine Position.

a *p* > 0.05 (coefficients not significantly different from zero).

## Discussion

Given the multifactorial nature of SDB, airway narrowing may be induced by obesity, adenotonsillar hypertrophy, retrognathia, or other factors that obstruct the airway.^12^, ^13^, ^19^ Patients with adequate weight may develop OSA due to craniofacial growth restriction, whereas obese patients typically present with soft tissue enlargement.

Craniofacial growth influenced by genetic inheritance and functional factors can impact on general health.^21^ Several studies prove that craniofacial morphology is a strong risk factor for pediatric SDB.^22^ Obstructive sleep apnea has been associated with deflections in craniofacial growth and development in children. A small maxilla or mandible can predispose the child to snore and obstructive sleep apnea.^12^, ^19^, ^21^ Orthodontic treatments such as RME,^21^, ^23^ orthopedic orthodontic appliances,^24^ and functional orthodontic appliances^25^ can reduce snoring and OSA in ideal conditions during child growth and development.^21^ Effective treatment for SDB should target one or more risk factors to help cure the obstruction. Therefore, an accurate diagnosis of the etiology of SDB is crucial for treatment success, which must be multifaceted for all existing factors.^19^

Patients with maxillary transverse deficiency are predisposed to OSA and often present with tooth crowding and malocclusion, which can be treated by RME.^26^ RME is believed to decrease nasal resistance and facilitate the passage of air through the nose. In addition to improving the quality of nasal breathing, RME transversally increases the maxilla and thus enhances the tongue position, allowing proper lip sealing when the mouth is closed.^23^, ^27^ It also indirectly increases the oropharyngeal space.

In a systematic review, Niu et al.^28^ studied the three-dimensional analyses of short- and long-term effects of rapid maxillary expansion on nasal cavity and upper airway. They concluded that there was an increase in the volume of the nasopharynx and oropharynx in the short term. However, more studies need to evidence these changes and the long-term stability of these results. Pirelli et al.^29^ confirmed the improvement of chronic snoring and OSA signs and symptoms with an increase in the total volume of the upper airways after RME in a recent study. We also confirmed these findings. In our research, the oropharynx volume in the total sample increased statistically significantly after RME compared to that observed before RME. As for the comparison between groups, the mean oropharyngeal volume was higher in the primary snoring group than in the OSA group; however, the differences were neither significant before nor after RME. Correlations values between saturation and oropharyngeal volume were not significant, both before RME and after RME. Based on the findings of the present investigation, it is not recommended to use volumetric changes of the airways after RME of children as a valid and reliable aspect to quantify breathing improvement. Most patients will show a degree of volumetric airway change that will not correlate to the quantitative breathing improvement.

Due to the complex nature of OSA, a multidisciplinary approach can optimize treatment. Although physicians are first-line providers in treatment and diagnosis, an integrated approach of health professionals including orthodontists, speech therapists, nutritionists, and psychologists can improve sleep-disordered breathing in childhood. Educating healthcare professionals about the potential benefits of RME is an essential step in interdisciplinary care. Assuming that craniofacial abnormalities are risk factors for SDB^12^ and can be corrected or minimized in a non-invasive manner only in childhood, new studies with larger samples are fundamental for preventing adult’s apnea.

Patients in this study will continue to be followed up to assess growth, development, and stability treatment benefits. In addition, complementary studies in association with other functional^25^ or fixed orthodontic appliances^24^ (for patients with skeletal class II) and myofunctional treatment^30^ to improve breathing and OSA should be considered to maintain or even improve the quality of life of these patients.

## Conclusion

This study showed that when rapid maxillary expansion is performed in individuals with SDBs, there were statistically significant differences in oropharyngeal volume between pre-RME and post-RME. However, no correlation was found between oropharyngeal volume increase and oxygen saturation values.

## Funding

This work was supported by the Coordenação de Aperfeiçoamento de Pessoal de Nível Superior (CAPES) [grant number: 8882.430440/2019-01], and by Associação Fundo de Incentivo à Pesquisa (AFIP).

## Conflicts of interest

The authors declare no conflicts of interest.

No conflicts of interest of any kind were involved in the study. The funding sources had no involvement for the conduct of the research, preparation of the article, study design, collection, and analysis, interpretation of data, writing, and decision to submit the article for publication.
